# The BLM helicase is a new therapeutic target in multiple myeloma involved in replication stress survival and drug resistance

**DOI:** 10.3389/fimmu.2022.983181

**Published:** 2022-12-09

**Authors:** Sara Ovejero, Elena Viziteu, Laure Dutrieux, Julie Devin, Yea-Lih Lin, Elina Alaterre, Michel Jourdan, Jihane Basbous, Guilhem Requirand, Nicolas Robert, Hugues de Boussac, Anja Seckinger, Dirk Hose, Laure Vincent, Charles Herbaux, Angelos Constantinou, Philippe Pasero, Jérôme Moreaux

**Affiliations:** ^1^ Department of Biological Hematology, CHU Montpellier, Montpellier, France; ^2^ Institute of Human Genetics, UMR 9002 CNRS-UM, Montpellier, France; ^3^ Diag2Tec, Montpellier, France; ^4^ Department of Hematology and Immunology, Vrije Universiteit Brussel (VUB), Brussels, Belgium; ^5^ Department of Clinical Hematology, CHU Montpellier, Montpellier, France; ^6^ Institut Universitaire de France (IUF), Paris, France

**Keywords:** multiple myeloma, BLM, DNA damage, replication stress, drug resistance

## Abstract

Multiple myeloma (MM) is a hematologic cancer characterized by accumulation of malignant plasma cells in the bone marrow. To date, no definitive cure exists for MM and resistance to current treatments is one of the major challenges of this disease. The DNA helicase BLM, whose depletion or mutation causes the cancer-prone Bloom’s syndrome (BS), is a central factor of DNA damage repair by homologous recombination (HR) and genomic stability maintenance. Using independent cohorts of MM patients, we identified that high expression of BLM is associated with a poor outcome with a significant enrichment in replication stress signature. We provide evidence that chemical inhibition of BLM by the small molecule ML216 in HMCLs (human myeloma cell lines) leads to cell cycle arrest and increases apoptosis, likely by accumulation of DNA damage. BLM inhibition synergizes with the alkylating agent melphalan to efficiently inhibit growth and promote cell death in HMCLs. Moreover, ML216 treatment re-sensitizes melphalan-resistant cell lines to this conventional therapeutic agent. Altogether, these data suggest that inhibition of BLM in combination with DNA damaging agents could be of therapeutic interest in the treatment of MM, especially in those patients with high BLM expression and/or resistance to melphalan.

## Introduction

Multiple myeloma (MM) is the second most common hematologic cancer after non-Hodgkin lymphoma. It mainly affects patients over 70 years of age and to date there is no definitive treatment. Treatments of choice include a combination of immunomodulatory drugs, proteasome inhibitors, DNA damaging agents, and monoclonal antibodies among others, together with autologous stem cell transplantation in transplant-eligible patients [reviewed in (1)]. Although these treatments can extend the life expectancy of the patients, eventually almost all of them develop resistance to chemotherapy and relapse. Therefore, MM remains a non-curable disease with significant morbidity and a median survival of 10 years for patients eligible to high dose melphalan (2–5). These facts point at an urgent need of better and targeted therapeutic approaches for MM patients to reduce the morbidity and overcome the resistance to current treatments.

At the cellular level, MM is characterized by the accumulation of malignant plasma cells, called multiple myeloma cells (MMCs), in the bone marrow (BM). These MMCs present high somatic hypermutation of immunoglobulin genes, characteristic aberrant chromosomal translocations (3, 6, 7), and a strong dependence on BM microenvironment, which provides survival signals and mediates drug resistance (8–10). Recent advances in treatment with the approval of several novel agents and their combinations have significantly improved patient outcome (11). However, patients invariably relapse after multiple lines of treatment, with shortened intervals between relapses, and finally become resistant to all treatments, resulting in loss of clinical control over the disease. MM is a genetically and clinically heterogeneous disease. Genome sequencing studies have revealed considerable heterogeneity and genomic instability, a complex mutational landscape and a branching pattern of clonal evolution (12). Epigenetics has also been shown to play a role in the disease progression and resistance to treatments, and deregulation of epigenetic factors, notably of those associated with DNA methylation, are related to bad prognosis in MM patients (13–15). In addition, intraclonal heterogeneity adds more complexity to MM pathophysiology and is most likely crucial for the progression of the disease and the relapse after treatment (16–19). In this context, genetic and epigenetic-wide screens constitute attractive strategies to understand the onset and development of MM as well as to identify new candidates to overcome drug resistance.

In our effort to identify new therapeutic targets in MM, we found that the *BLM* gene, which encodes the Bloom’s syndrome (BS) protein BLM, an ATP-dependent 3’-5’ DNA helicase (20–22), is associated with a poor prognostic value in MM patients. Moreover, we have recently reported that BLM has a role in the regulation of cell proliferation and survival during human normal B to plasma cell (PC) differentiation (23). BLM belongs to the highly evolutionary conserved RECQ family of DNA helicases; four of the five human genes of this family, BLM, WRN, RECQL4 and RECQL1, are associated with inheritable premature-aging and cancer-prone diseases (22, 24–26). The mutations of BLM disrupting its ATPase or helicase activity cause BS (27), a rare autosomal recessive genetic disorder characterized by developmental problems, growth retardation, immunodeficiency, sunlight sensitivity, fertility defects, and cancer predisposition associated with genomic and chromosomic instability (28–30). Soon after BS was first described, it was reported that lymphocytes from BS patients present a large increase in sister chromatid exchanges, which to date remains one of the cellular hallmarks of the disease (29, 31, 32). This cellular phenotype is due to the role of BLM in preventing sister chromatid and homolog chromosome exchanges during homologous recombination (HR) (33). Indeed, BS patients present a high sensitivity to DNA damaging agents commonly used in chemotherapy because the loss of BLM activity causes deficient DNA repair (34, 35). In particular, BLM is necessary for normal replication completion and is involved in several steps of the HR process. First, BLM is recruited to DNA double strand breaks (DSBs) in a manner dependent on the presence of NBS1, MRE11 and ATM. ATM activity is essential only for the early recruitment of BLM, whereas polyubiquitination of BLM and its subsequent interaction with NBS1 are required for its retention at DSBs (35). Then, BLM together with the endonuclease DNA2 is involved in 5’-end DNA resection during the initiation step of DSBs repair by HR (21, 36, 37). Later, once DNA synthesis has been completed across the DNA break, BLM resolves the Holliday junctions to restore the separated DNA duplexes (38–40). However, BLM can also have anti-recombinogenic activity by disrupting the D-loops formed during the strand invasion step of HR, and other studies have underlined the dual role of BLM through its pro- and anti-recombinogenic activities to balance HR and non-homologous end joining (NHEJ) in order to preserve genome stability (34, 41–45). BLM also maintains genome stability at stalled replication forks by promoting fork regression and restart (46–48), resolves mitotic chromosome bridges (49–51), and participates in telomere maintenance by resolving G-quadruplexes that can interfere with telomere replication (52–54).

As most cancers, MM and other hematological malignancies are also characterized by genomic instability that may arise from defective DNA replication and repair pathways (55–57). It has been highlighted that a subgroup of MM patients displays high chromosomal instability and replication stress which correlate with poor outcome (58–60). Along this line, we previously reported the importance of RECQ1 helicase in the survival to replication stress and drug resistance of MM cells (61, 62). BLM helicase, which belongs to the same family as RECQ1, is crucial in the maintenance of chromosomal stability and has been clearly associated with cancer development in BS patients. Here, we report that *BLM* expression is deregulated in several MM patient cohorts and that its overexpression is associated with poor prognosis. A novel BLM inhibitor, ML216, is a small molecule that inhibits the catalytic activity of BLM more than other RECQ family helicases (63, 64). ML216 has been used to characterize BLM function in HR (35), but its potential as an anti-cancer therapy has barely been addressed and to date no available studies have evaluated it in the context of MM.

Here, we characterized the importance of BLM for MM pathophysiology and resistance to treatments as well as the use of ML216 alone and in combination with current MM chemotherapies. Using a unique collection of human MM cell lines (HMCLs) that recapitulate the heterogeneity and complexity of MM patients (65, 66), we found that different HMCLs display different sensitivity to BLM inhibition by ML216. Characterizing the impact of BLM inhibition on MM plasma cell survival, we found that ML216 induces DNA damage and apoptosis. Moreover, co-treatment of HMCLs with ML216 and melphalan, a common anti-myeloma drug, has a synergistic effect leading to increased MMC death. Our results suggest that BLM inhibition in combination with melphalan could be of therapeutic interest in the treatment of MM.

## Materials and methods

### BLM expression analysis and gene set enrichment analysis

Patients’ MMCs were purified using anti-CD138 MACS microbeads (Miltenyi Biotec, Bergisch Gladbach, Germany) and their gene expression profile (GEP) obtained using Affymetrix U133 plus 2.0 microarrays as described (Array Express public database [E-MTAB-372]) (67). Publicly available cohorts of newly-diagnosed MM patients treated with high dose melphalan and autologous hematopoietic stem cell transplantation (UAMS-TT2 and TT3 (GSE24080), and Hovon (GSE19784) cohorts) were also used. Gene expression data were normalized with the MAS5 algorithm and analyses processed with GenomicScape (http://www.genomicscape.com) (68). Gene Set Expression Analysis (GSEA) was used to identify genes and pathways differentially expressed between populations. Difference in overall survival between groups of patients was assayed with a log-rank test and survival curves plotted using the Kaplan–Meier method (Maxstat R package) (69).

### Human myeloma cell lines and drug treatments

XG1, XG2, XG7, XG12, XG19 and XG21 HMCLs are IL-6 dependent cell lines obtained as previously described (65). Upon removal of IL-6, these cell lines progressively apoptose within 10 to 14 days and are routinely maintained in RPMI 1640 GlutaMAX medium (61870044, Gibco) supplemented with 10% fetal calf serum (CVFSVF00 01, Eurobio) and with IL-6 (2 ng/ml) (65). AMO-1, LP1 and OPM2 were purchased from DSMZ (Braunsweig, Germany) and RPMI8226 from ATCC (Rockville, MD, USA). These cell lines were grown in RPMI 1640 GlutaMAX medium (61870044, Gibco) supplemented with 10% fetal calf serum (CVFSVF00 01, Eurobio). Melphalan-resistant XG2 and XG7 cell lines were derived from XG2 and XG7 parental cell lines after sequential *in vitro* treatment and selection (70). HMCLs were authenticated according to their short tandem repeat profiling and their gene expression profiling using Affymetrix U133 plus 2.0 microarrays deposited in the ArrayExpress public database under accession numbers E-TABM-937 and E-TABM-1088. Whole exome sequencing analysis was performed on XG2 and XG7 melphalan-resistant cell lines and the corresponding parental cell lines as previously reported (66). The WES library preparation was done with 1000 ng of input DNA. Sequences of exome were enriched using SureSelect^xt^ kit and SureSelect^xt^ All Exons v5 library (Agilent Technologies, Santa Clara, California, USA). Paired-end exome sequencing was performed on the enriched exome sequences using the illumina NextSeq500 sequencing instrument (Helixio, Clermont-Ferrand, France), generating 75 bp paired-end reads with 100X average coverage per sample.

Drugs used in this study: ML216 (SML0661, Sigma) and melphalan (Y0001457, European Pharmacopoeia Reference Standard).

### Generation of XG2 cells with BLM knock-down

XG2 cells were transduced with control or BLM miRNA lentiviral particles. BLM miRNAs (Invitrogen, Carlsbad, USA) were cloned in the pLenti4-EZ-mIR plasmid (Invitrogen) as described (61). This plasmid contains the shRNA sequence and also the *GFP* gene under the control of Tet operators. Cells were selected with 12.5 μg/ml of zeocin for 2 weeks. When selection was completed, cells were maintained in the presence of 6.25 μg/ml zeocin to keep the selection pressure. Before every experiment, zeocin was removed from the medium by extensive washing and cells were plated in zeocin-free fresh medium. BLM depletion was validated by western blot using anti-BLM (ab476, Abcam).

### Evaluation of ML216 toxicity on primary multiple myeloma cells

Bone marrow samples from untreated MM patients (n = 7) were obtained at the University Hospital of Montpellier after patients’ written informed consent in accordance with the Declaration of Helsinki and agreement of the Montpellier University Hospital Centre for Biological Resources (DC-2008-417). Bone marrow mononuclear cells are cultured with IL-6 (2ng/ml) (61, 71) seeded at 5x10^5^cells/mL in RPMI 1640 medium, 5% FCS, 2ng/mL IL-6, and cultured with or without ML216 (3 μM, 6 μM or 10 μM) for 4 days as described (61, 71). In each culture group, viability and cell count were assayed and MM cell cytotoxicity was assessed by flow cytometry (61, 71). MM plasma cells (CD138+) were detected using anti-CD138-phycoerythrin monoclonal antibody (Immunotech, Marseille, France) and all CD138- cells were analyzed as non-myeloma cells.

### Proliferation assays and synergy matrixes

For IC50 determination, HMCLs were seeded at 10000 cells/well and cultured for 4 days in 96-well flat-bottom plates in presence of ML216 at concentrations ranging from 0.78 μM to 100 μM. Cell proliferation was evaluated using CellTiter-Glo (CTG) Luminiscent Assay (G7573, Promega) according to manufacturer’s protocol and luminescence was measured using a Centro LB 960 luminometer (Berthold Technologies, Bad Wildbad, Germany). IC50 for each HMCL was calculated using non-linear regression analysis in GraphPrism software.

For evaluation of ML216 and melphalan synergy, increasing concentrations of each single drug were combined with all concentrations of the other drug so all possible combinations were evaluated. Cell growth was evaluated with CTG reagent as described above. For each combination, the percentage of expected growing cells in the case of effect independence was calculated with Bliss equation using R package “SynergyFinder”.

### Apoptosis and cell cycle analysis

Cells were treated with the indicated concentrations of ML216 and melphalan for 48 and 96h. Cells were collected, counted and 10^5^ cells per condition were processed with the Annexin V kit (556421, BD Biosciences) according to manufacturer’s instructions. Apoptotic cells (AnnexinV+) were quantified by flow cytometry.

For cell cycle analysis, cells were cultured and treated as described above. To mark replicating cells, culture medium was supplemented with 10 μg/ml BrdU (bromodeoxyuridine) during the last hour of each treatment and samples were processed with the APC BrdU flow kit (552598, BD Biosciences) according to manufacturer’s instructions. Cells cycle phases were analyzed by flow cytometry. BrdU+ cells were assigned to S-phase and BrdU- cells were classified as G0/G1 or G2/M phases based on their DNA content.

All flow cytometry acquisitions were done on a Fortessa flow cytometer (BD biosciences) and quantifications were done with Kaluza software.

### Western blot

For comparison of BLM protein levels in HMCLs, cells were lysed with RIPA buffer (sc-24948, Santa Cruz) supplemented with halt protease and phosphatase inhibitor cocktail (78442, Thermo Scientific). Cell extracts were quantified by BCA assay (23225, Thermo Scientific), absorbance at 570 nm was measured using a spectrophotometer (Tecan) and a linear regression was performed to determine protein concentration in each sample. Typically, 20 μg of protein extract were loaded per sample in 8% polyacrylamide gels.

For analysis of apoptotic and DDR pathways, 1 million cells were directly lysed in 300 μl of Laemmli buffer (1x), vortexed, boiled at 95°C for 5 minutes and 20 μl were loaded per sample in 8%, 10% and 14% polyacrylamide gels.

Antibodies used in this study are: anti-BLM (ab476, Abcam), anti-WRN (4666, Cell Signaling), anti-RECQ1 (ab22830, Abcam), anti-RECQL5 (sc-515050, Santa Cruz), anti-Tubulin (2144S, Cell Signaling), anti-pSer15_p53 (9284S, Cell Signaling), anti-p53 (9282S, Cell Signaling), anti-p21 (2946S, Cell Signaling), anti-p27 (3688S, Cell Signaling), anti-γH2AX (05-636, Millipore), anti-PARP (9532S, Cell Signaling), anti-pThr68_Chk2 (2197S, Cell Signaling), anti-Chk2 (2662, Cell Signaling), anti-pSer345_Chk1 (2348S, Cell Signaling), anti-Chk1 (2345S, Cell Signaling), anti-Caspase 3 (9662S, Cell Signaling), anti-Caspase 8 (9746, Cell Signaling), anti-Caspase 9 (9502S, Cell Signaling).

### Immunofluorescence

Cells were deposited on poly-lysine coated slides (J2800AMNZ, Thermo Scientific) using a Cytospin 4 centrifuge (Thermo Scientific) at 600 rpm for 10 minutes. Soluble cell fraction was pre-extracted by incubation with cold cytoskeleton buffer (CSK: 10 mM PIPES, pH 7, 100 mM NaCl, 300 mM sucrose, 3 mM MgCl2, 0.7% Triton X-100) (2 x 3 minutes), fixed with 4% PFA-PBS, and saturated with 3% BSA-PBS for 1h at RT. Anti-BLM antibodies (ab476, Abcam; sc-365753, Santa Cruz) were diluted at 1:200 and anti-nucleolin (ab22758, Abcam) was diluted at 1:1000 in saturation buffer and incubated on slides in a humid chamber for 90 minutes. Slides were washed 3 x 5 minutes with PBS-0.01% Tween, incubated protected from light in a humid chamber with secondary antibody (A11008, Invitrogen) 1:500 for 45 minutes at RT. Washed again 3 x 5 minutes with PBS-0.01% Tween, incubated with DAPI (20 μg/ml) in H_2_O for 5 minutes and washed 3 times with H_2_O. Slides were air dried and mounted with Prolong Gold (P36930, Invitrogen) and let to dry overnight. Image acquisition was performed with a ZEISS Axio Imager Z1 Apotome microscope and analysis was done with Omero server.

## Results

### *BLM* expression is associated with a poor outcome in multiple myeloma

**Figure 1:**
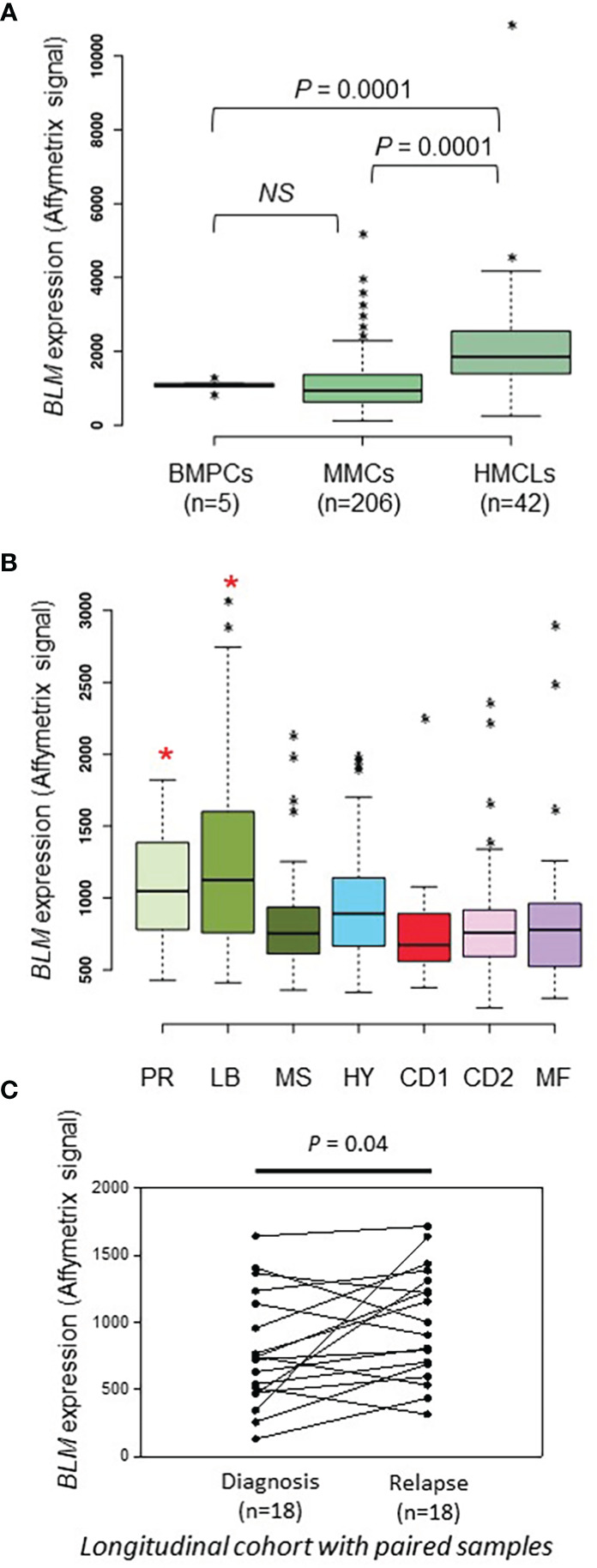
BLM expression in MM. **(A)** *BLM* expression analysis by Affimetrix microarrays from normal BMPCs from 5 healthy donors (median: 1133; range: 863-1328), MMCs from 206 MM patients from the HM cohort (median: 935; range: 113-5206), and 42 HMCLs (median: 1848; range: 246-10901). * marks outliers. Statistical analysis used a Student’s t-test of paired samples. **(B)** Gene expression profiling of MMCs of the patients of UAMS-TT2 cohort were used. Patients (n = 250) were classified in the 7 molecular groups of MM. PR: cell cycle and proliferation, LB: low bone disease, MSET: MMSET overexpression, HY: hyperdiploid signature, CDNN1: Cyclin D1 overexpression, CDNN2: Cyclin D2 overexpression, MAF: overexpression of MAF and MAFB genes. Small asterisks mark outliers in each group. Big red asterisks indicate that *BLM* expression is significantly higher in the group compared to all the patients of the cohort (P < 0.05) (Student’s t-test). **(C)** BLM expression is significantly higher at relapse compared to diagnosis in a longitudinal cohort of 18 paired patient’s samples (paired Student’s t-test). * p-value < 0.05.

We previously reported that *BLM* gene had a bad prognostic value in the Heidelberg-Montpellier (HM) MM cohort of patients (58) and that *BLM* expression was significantly upregulated in MM according to bioinformatics analysis of one publicly available cohort of MM patients with gene expression dataset (62). Therefore, we further studied *BLM* in the pathophysiology of MM and validate our previous observations with several independent cohorts of patients. No significant difference in *BLM* expression between normal bone marrow plasma cells (BMPCs; n=5; median: 1133; range: 863-1328) and MMCs from patients (n=206; median: 935; range: 113-5206) was found (**Figure 1A**). Furthermore, although we did not observe a statistical difference with BMPCs, *BLM* mRNA levels appeared heterogeneous in MMCs ranging from 236 to 3079 in Affymetrix signal (**Figure 1A**, MMCs outliers marked by *). However, *BLM* expression was significantly higher in HMCLs (n=42; median: 1848; range: 246-10901) when compared to both normal BMPCs and primary MMCs (P = 0.0001) (**Figure 1A** and **Supplementary Figure 1A**), suggesting an increase in *BLM* levels with the progression of the disease. Primary MMCs of untreated patients can be classified into seven molecular groups associated with different patient survival (72). According to this classification, *BLM* expression was significantly higher in the poor prognosis “proliferation” subgroup (PR; P < 0.5) and “low bone disease” subgroup (LB; P < 0.01) (**Figure 1B**). Finally, we investigated the *BLM* expression in a cohort of 18 patients with paired samples at diagnosis and relapse, and identified a significant higher expression of *BLM* at relapse (P=0.04) (**Figure 1C**).

**Figure 2:**
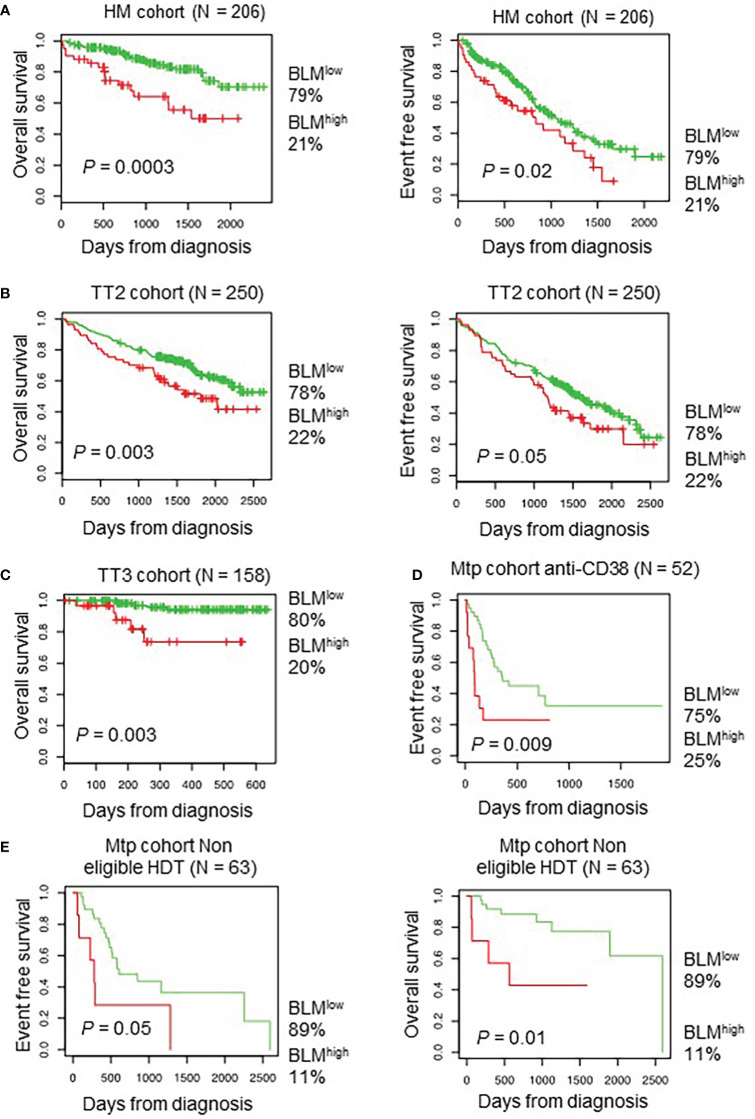
High *BLM* expression is associated with a poor outcome in MM. Correlation between *BLM* expression and overall survival was analyzed using Maxstat R package in independent cohorts of MM patients: including patients at diagnosis treated by HDT and ASCT (**A**, left panel) HM cohort; (**B**, left panel) UAMS-TT2 cohort; **(C)** TT3 cohort; a cohort of patients at relapse treated by Anti-CD38 antibody (Daratumumab) **(D)** Mtp cohort anti-CD38 and a cohort of patients at diagnosis non eligible to HDT and ASCT **(E)** Mtp cohort non eligible HDT. (**A, B**, right panels) High expression of *BLM* is associated with shorter event free survival in the HM, TT2 and Mtp cohort non eligible HDT cohorts.

Using Maxstat R package (69), we determined that high *BLM* expression in MMCs could predict shorter overall survival (OS) in four independent cohorts of previously untreated patients that were homogenously treated with high melphalan dose (HDT) followed by autologous stem cell transplantation (ASCT), a standard-of-care therapy for newly diagnosed MM (73) (HM cohort (n = 206): P = 0.003; UAMS-TT2 cohort (n = 250): P = 0.0002; TT3 cohort (n = 158): P = 0.0008; Hovon cohort (n = 282): P = 0.04) (**Figures 2A–C**). A high *BLM* expression could also predict for shorter event free survival (EFS) in the HM and TT2 cohorts (**Figures 2A, B**, right panels). In a COX multivariate analysis [including ISS, B2M level, t (4, 14), del17p, Gep70 score (74), IFM score (75), Growth Proliferation Index (GPI) (67) and RS (76)], BLM expression, B2M level, t (4, 14) and RS remain independent prognostic factors (**Supplementary Table S1**). In addition, Gene Set Enrichment Analysis (GSEA) showed that patients with high *BLM* expression level and high-risk present a significant enrichment of proliferation-associated genes (Reactome cell cycle and mitotic: P = 0.01; reactome G1/S transition: P = 0.01) (**Supplementary Figure S1B**). However, no significant correlation between *BLM* expression and MMC plasma cell labeling index (77) was found (**Supplementary Figure S1C**). Furthermore, no significant difference was identified comparing GPI subgroups (**Supplementary Figure S2A**). BLM expression was significantly higher in high-risk MM patients defined by RS score (76) (**Supplementary Figure S2A**). (High *BLM* expression was also associated with a poor outcome in a cohort of MM patients at relapse treated by anti-CD38 MoAb (78) (**Figure 2D**) at diagnosis non eligible to HDT and ASCT (n=63) (**Figure 2E**). These data indicate that high *BLM* expression is associated with poor prognosis in MM patients and correlates with increased expression of cell cycle progression genes, even though cells with high *BLM* expression do not show increased proliferation.

### BLM inhibition in MMCs affects proliferation and induces apoptosis

**Figure 3:**
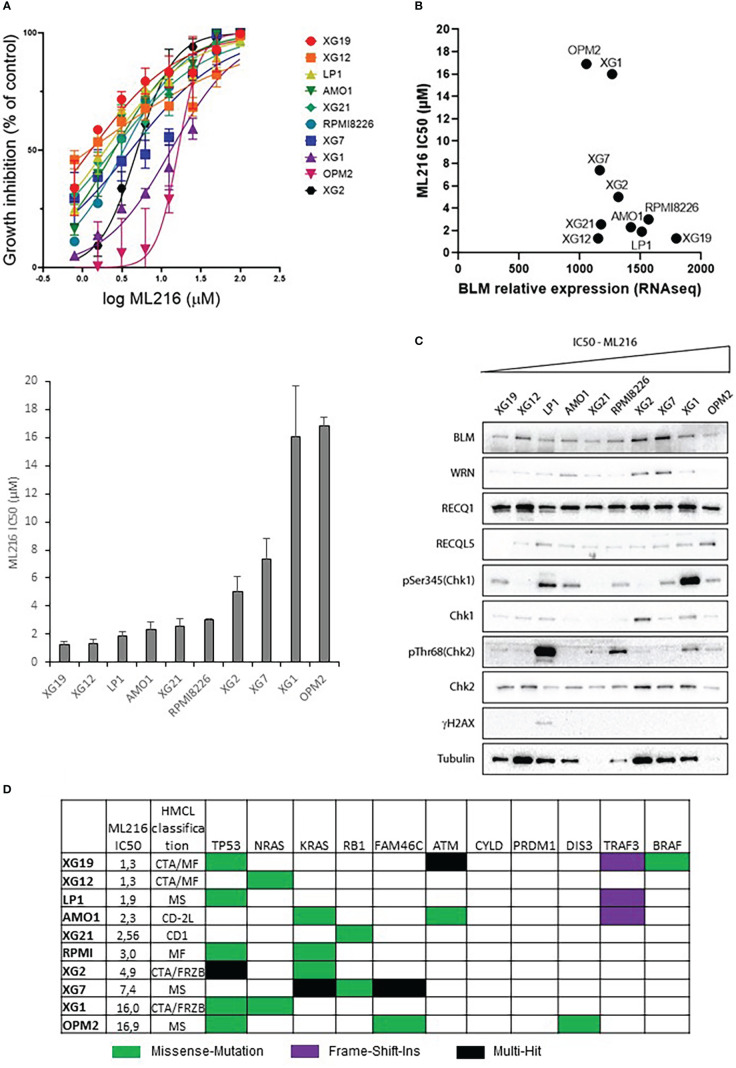
Effect of ML216 in HMCLs. **(A)** 10 HMCLs were treated with increasing doses of ML216 (0.78 – 100 μM). At day 4, cell viability was assessed using CellTiter-Glo Luminiscent Cell Viability Assay. IC50 for each cell line was calculated using GraphPrism software. Upper panel shows the non-linear regression curve of growth inhibition for all HMCLs. Low panel shows the IC50 value for each HMCL. Data are based on at least 3 independent experiments. **(B)** *BLM* expression and sensitivity to ML216 (IC50) do not correlate after applying a Spearman’s test. **(C)** Western blot analysis of the protein level of the RECQ helicases and basal DDR markers in 10 HMCLs. Cell lines are ranked from left to right in order of increasing IC50 for ML216. Note that in most cell lines protein levels do not correlate with *BLM* expression levels determined by RNAseq in Figure 3B. **(D)** Mutational status of MM frequently mutated genes in the 10 HMCLs used for the other experiments in this figure. “HMCL classification” refers to the MM molecular group classification. PR (cell cycle and proliferation), LB (low bone disease), MSET (aberrant expression of FGFR3 and MMSET genes), HY (hyperdiploid signature), CDNN1 (overexpression of Cyclin D1), CDNN2 (overexpression of Cyclin D2), and MAF (overexpression of MAF and MAFB genes) (72). Data extracted from (66).

ML216 is a small molecule that inhibits BLM DNA unwinding activity by blocking its nucleic acid binding site. This inhibitor has been shown to be selective for BLM over other members of the RECQ family *in vivo* (63, 64). Hence, the response to ML216 was tested in a panel of ten HMCLs representative in part of the molecular heterogeneity of MM (65). Treatment with this drug inhibited cell proliferation in a dose-dependent manner with a median IC50 of 2.78 µM (range: 1.2-16.9 µM) (**Figure 3A**). RNA-seq and western blot analysis showed marked differences in BLM expression among HMCLs (**Figures 3B, C**). However, there was not any correlation between *BLM* gene expression or BLM protein levels and HMCLs sensitivity to ML216. Importantly, BLM was not mutated in any of these HMCLs according to our sequencing data [data not shown published in (66)]. The differences in the protein levels of the RECQ helicases WRN, RECQ1 and RECQL5, and the basal level of DDR activation were also not correlated with the sensitivity to ML216 (**Figure 3C**), thus ruling out that the effect of the BLM inhibitor on cell proliferation was due to a non-specific effect on another helicase or a higher level of basal DNA damage specific to certain cell lines. Additionally, we did not find any correlation between the sensitivity to the BLM inhibitor and the MM molecular subgroups (65), nor the recurrent mutations reported in MM, nor mutations in genes involved in the DDR (66, 79) (**Figure 3D**). However, our sequencing data identified in each cell line mutations in numerous genes involved in transcription (66). Therefore, it is possible that deregulation of transcription may have an indirect impact in the DDR and other molecular pathways, making some cells more vulnerable to BLM inhibition than others. In addition, BLM is involved in other cellular processes other than DNA repair, such as replication, telomeric maintenance or ribosomal DNA regulation among others (80), which suggests that differences in the regulation of these processes may also account for the differential sensitivity of the cell lines to ML216.

ML216 has been described to exclusively inhibit BLM unwinding activity without any further impact on its biological regulation (63, 64). To confirm that this is the case in MM cells, we analyzed BLM protein levels upon ML216 treatment in the sensitive cell lines XG2 and XG19. The levels of BLM and the RECQ helicases WRN, RECQ1 and RECQL5 did not significantly change after 48h and 96h in presence of ML216 (**Supplementary Figure S3A**). BLM is a nuclear protein recruited to chromatin in response to DNA damage. It can form foci and micro-speckles (81) and also localizes to the nucleolus (82). To determine the subcellular localization of BLM in HMCLs, XG19 and XG2 cells were treated with ML216 for 48h, soluble proteins were pre-extracted by cytoskeleton (CSK) buffer prior to fixation and immunofluorescence detection. Chromatin bound BLM localized to the nucleolus and nuclear foci in basal conditions in XG19 and XG2 cells and no significant change was observed upon ML216 treatment (**Figure S3B**). Intriguingly, in XG2 cells ML216 treatment induce a partial relocalisation of nucleolin in the chromatin, and a conformational change of the nucleoli, which appeared more elongated (**Figure S3B**, right), although BLM remained localized in them. Together, these data indicate that the chemical inhibition by ML216 does not affect BLM levels or localization in MM cells, and that the observed cytotoxicity is likely due to the inhibition of BLM helicase activity.

**Figure 4:**
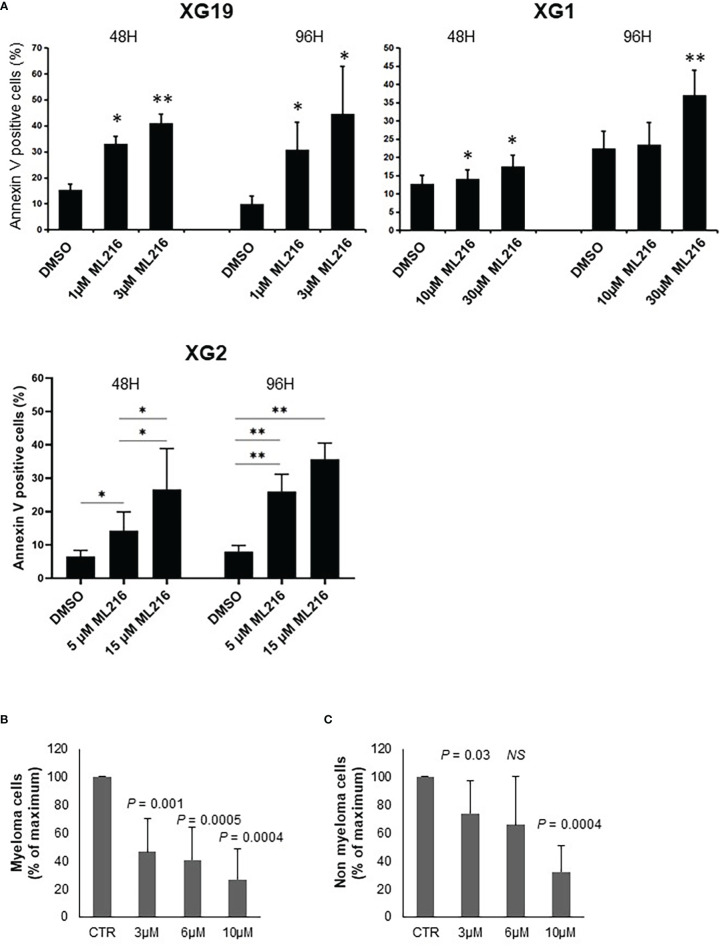
ML216 selectively induces apoptosis in MMCs. **(A)** XG19, XG2 and XG1 cell lines were treated for 48 and 96 hours with the indicated concentrations of ML216. Apoptotic cells were detected as Annexin V+ cells by flow cytometry. Results are the average of 3 independent experiments. * indicates a significant increase in apoptosis compared to DMSO controls using a Student’s t-test. **(B, C)** BM cells extracted from 7 MM patients were cultured with recombinant IL-6 and 3, 6 or 10 μM ML216 for 96 hours. Cytotoxicity was assessed by flow cytometry. **(B)** MM plasma cells (CD138+) were detected using an anti-CD138 antibody and **(C)** all CD138- cells were analyzed as non-myeloma cells. Number of cells in each condition was normalized with respect to the control. P-values indicate the significance of the observed differences after applying a Wilcoxon test for pairs. NS, not significant. * p-value < 0.05, ** p-value < 0.01.

In order to study the effect of BLM inhibition on MM cell survival, one ML216-resistant (XG1; IC50 = 13.2 µM) and two ML216-sensitive (XG19 IC50 = 1.2 µM; XG2 IC50 = 4.9 µM) HMCLs were chosen. ML216 induced marked apoptosis at all doses in the sensitive XG19 and XG2 cell lines already at 48 hours of treatment, whereas this effect was only mild on the resistant XG1 cell line even at 96 hours (**Figure 4A**). To confirm that BLM is also required for the survival of primary MMCs from patients, BM samples containing malignant MMCs with their BM environment and recombinant IL-6 were cultured with ML216 as described (13, 70, 83). ML216 treatment significantly reduced the median number of viable myeloma cells by more than 50% (P < 0.001; N = 7) compared to untreated control (**Figure 4B**). Of interest, the normal BM non-myeloma cells were less affected by ML216 treatment at low and mild doses (**Figure 4C**). We therefore conclude that viability of MM cell lines and primary MM cells depends on high BLM expression levels.

**Figure 5:**
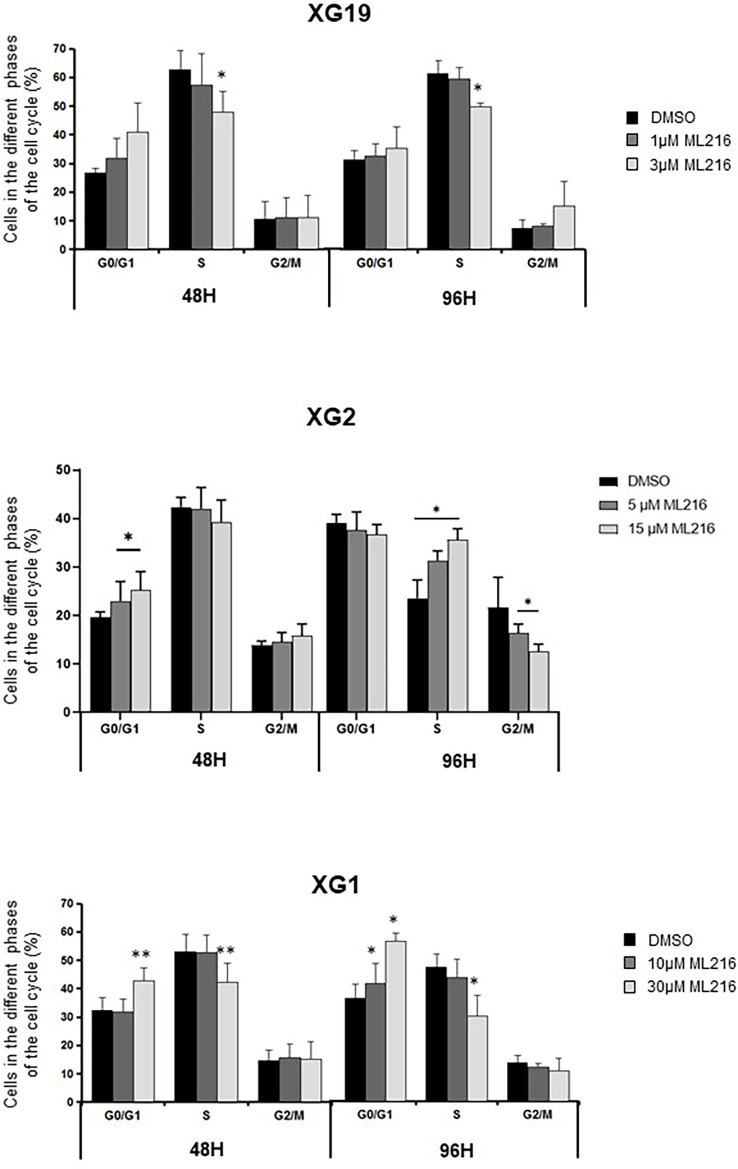
ML216 effect on cell cycle distribution in HMCLs. XG19, XG2 and XG1 cell lines were treated for 48 and 96 hours with the indicated concentrations of ML216. BrdU (10 μg/ml) was added during the last 1.5 hours of treatment. Cells were fixed and processed to detect BrdU incorporation and total DNA (see Materials and Methods for more details). BrdU+ cells were assigned to S-phase. BrdU- cells were assigned to G0/G1 or G2/M phases based on their DNA content. * indicates a significant difference compared to DMSO treated (control) cells after applying a Student’s t-test for pairs. Results are the mean of 3 independent experiments. ** p-value < 0.01.

BLM is a DNA helicase that unwinds DNA resulting from HR-mediated processes occurring during DNA replication and repair, such as D-loops (34, 41–45) or Holliday junctions (38–40). Therefore, a higher sensitivity of replicative or post-replicative cells to ML216 treatment was predicted. To address this possibility, we investigated the cell-cycle distribution of HMCLs treated with ML216 by flow cytometry. In the case of the XG1 cell line, ML216 induced a decrease in the percentage of cells in S-phase concomitant with an increase in G0/G1, indicative of a cell cycle arrest at the G1/S transition (**Figure 5**), and correlated also with more Annexin V positive cells (**Figure 4A**). In the XG19 sensitive cell line, ML216 treatment caused a decrease in the S-phase population (**Figure 5**) that correlated with an increase in the apoptotic population (**Figure 4A**) and no change was observed in the other phases of the cell cycle. In the XG2 cell line, ML216 treatment induced an accumulation in S-phase correlated to a decrease in the G2/M cell population (**Figure 4A**).Together, these data indicate that ML216 prevents entry and progression through S-phase in myeloma cells, causing an alteration of the cell cycle distribution. When cell cycle arrest due to ML216 treatment is sustained over time, apoptosis is triggered in all of the cell lines, suggesting that permanent inhibition of BLM causes DNA damage to a level that is lethal for myeloma cells.

### ML216-mediated inhibition of BLM synergizes with melphalan in MM

**Figure 6:**
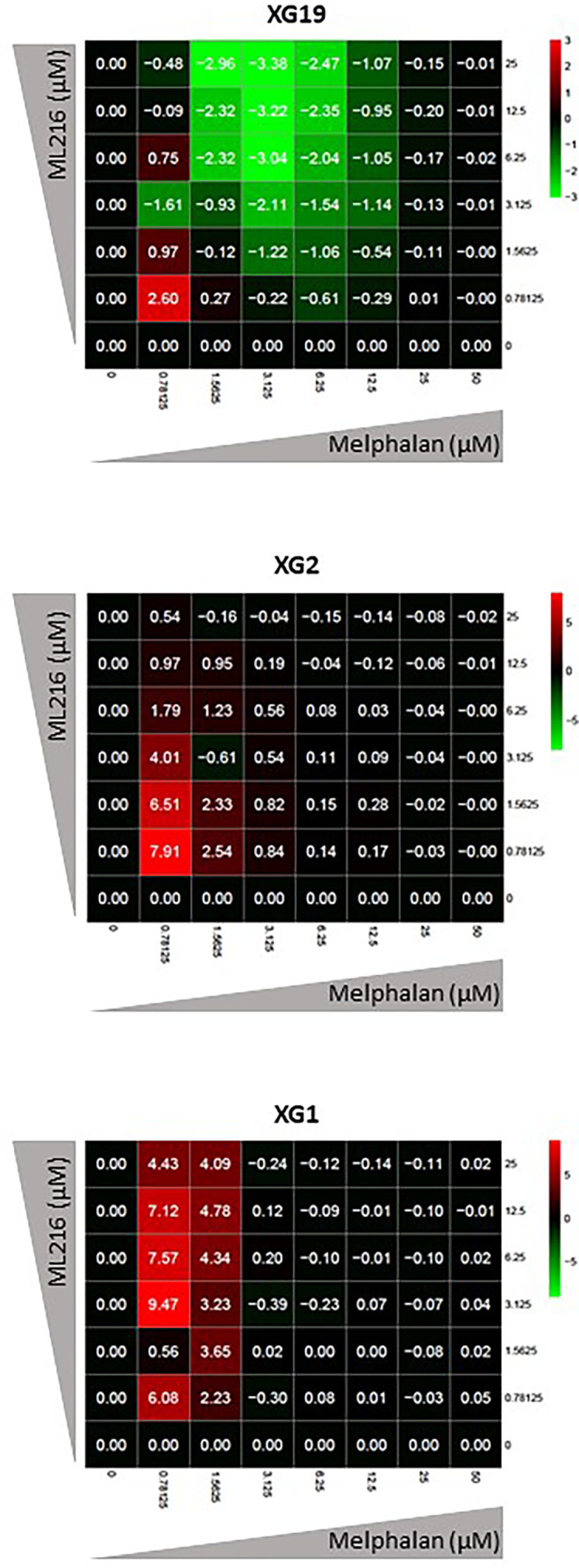
ML216 treatment synergizes with melphalan to inhibit HMCLs proliferation. Dose-response matrixes to measure synergy of ML216 and Melphalan co-treatment. Synergy scores are shown using a continuous pseudo-color scale ranging from bright-green (=antagonism) to bright-red (=synergism). XG19, XG2 and XG1 were treated with increasing concentrations of ML216 (0.78125 – 25 μM), and of the alkylating agent melphalan (0.78125 – 50 μM), for 4 days. Cell viability was assessed using the CellTiter-Glo Luminiscent Cell Viability Assay and was normalized to untreated conditions. Matrixes show the average of 3-4 independent experiments.

**Figure 7:**
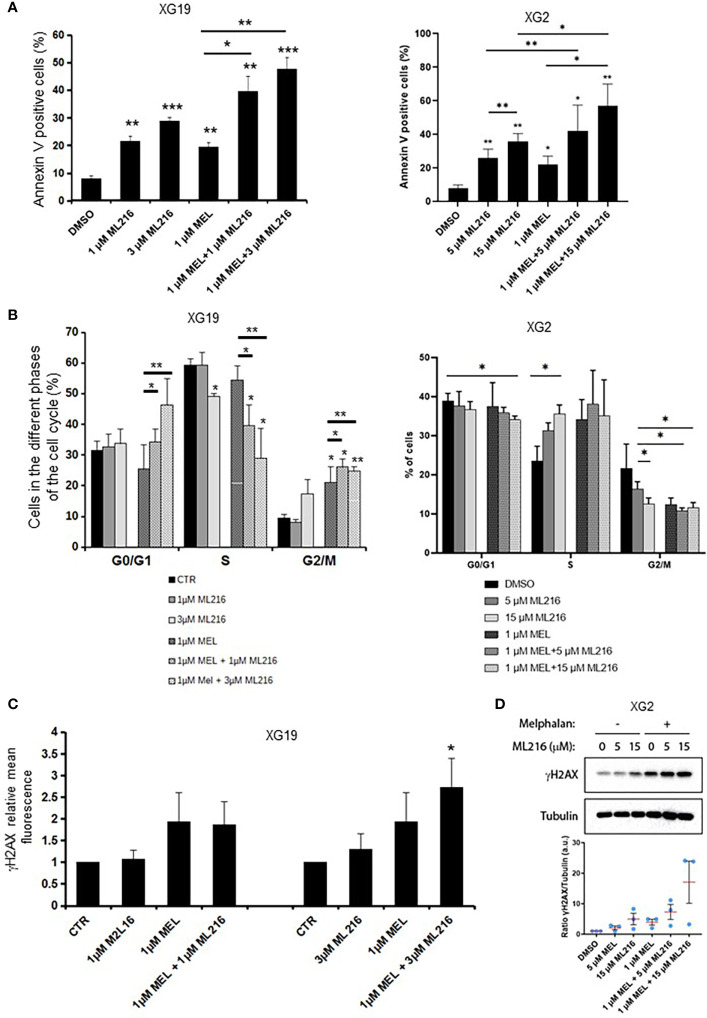
Effect of combination of ML216 and melphalan. **(A)** XG19 and XG2 cells were treated with ML216 and melphalan as indicated for 96 hours. Apoptotic cells were detected as Annexin V+ cells by flow cytometry. Results are the average of 3 independent experiments. * indicates a significant increase in apoptosis compared to DMSO controls using a Student’s t-test. **(B)** XG19 and XG2 cells were treated for 96 hours with the indicated concentrations of ML216 and melphalan. BrdU (10 μg/ml) was added during the last 1.5 hours of treatment. Cells were fixed and processed to detect BrdU incorporation and total DNA (see Materials and Methods for more details). BrdU+ cells were assigned to S-phase. BrdU- cells were assigned to G0/G1 or G2/M phases based on their DNA content. Asterisks indicate a significant difference compared to DMSO treated (control) cells after applying a Student’s t-test: * p-value < 0.05, ** p-value < 0.01. Results are the mean of 3 independent experiments. **(C)** XG19 cells treated as in **(B)** were processed to quantify γH2AX intensity by flow cytometry (see Materials and Methods for more details). * indicate a significant difference compared to DMSO treated (control) cells after applying a Student’s t-test for pairs: p-value < 0.05. **(D)** XG2 cells were treated with the indicated doses of ML216 and 1 μM melphalan for 48 hours. Cells were harvested and γH2AX levels were analyzed by western blot. One representative experiment out of three is shown. The graph shows the quantification of the γH2AX signal (a.u.: arbitrary units) with respect to tubulin, normalized to the control condition (DMSO) in 3 independent experiments. *** p-value < 0.001.

Various chemotherapeutic drugs are currently used to treat MM patients, and their rational and combinatorial use has proven to be a good treatment strategy to improve MM patient survival [reviewed in (1)]. Since another RECQ helicase was associated with resistance to genotoxic agents in MM (61), we next investigated whether ML216 could synergize with conventional MM treatment including melphalan, lenalidomide, and bortezomib. No synergy of ML216 with bortezomib (**Supplementary Figure S4A**), whereas a synergy between ML216 and lenalidomide was observed in XG2 and to a lesser extent in XG19 (**Supplementary Figure S4B**). However, ML216 treatment of XG2 did not induce any change in the protein levels of Myc and IRF4 (84) (**Supplementary Figure S4C**), and the molecular mechanism of this potential synergy remains to be explored. Interestingly, treatment with ML216 mildly potentiated the cytotoxic effect of melphalan in the XG1 and XG2 cell lines (**Figure 6**), whereas it had no major effect in the XG19 cell line (**Figure 6**). Since melphalan generates DNA damage and BLM is involved in DNA double-strand breaks (DSBs) resolution, we hypothesized that co-treatment with both drugs may increase DNA damage above a threshold that myeloma cells cannot cope with, leading to cell death. Indeed, combination of ML216 and melphalan induced a significant increase in apoptosis (**Figure 7A**), and affected cell cycle distribution inducing a decrease in the fraction of S-phase cells and an increase in G0/G1 cells (**Figure 7B**). As already mentioned, BLM promotes the HR-mediated repair of DNA DSBs (21, 36, 37). DSBs are marked by the presence of γH2AX, resulting from the phosphorylation of the H2AX histone variant on Ser139 by the checkpoint apical kinases ATM and ATR (85–87). Then, MDC1 (mediator of DNA damage checkpoint protein 1) binds to γH2AX and together orchestrate the recruitment of downstream DNA repair factors such as BRCA1, 53BP1, the MRN complex or RAD51 among others (88–90). Interestingly, the effect on cell cycle and cell death of ML216 and melphalan co-treatment (**Figures 7A, B**) correlated with an increase in γH2AX positive cells (**Figures 7C, D**), indicative of higher DNA damage levels in the presence of both drugs.

In order to further investigate the molecular mechanism at the origin of this synergism, we next analyzed the activation of apoptosis pathways in HMCLs treated with ML216 and melphalan by western blot. BLM inhibition in combination with melphalan induced PARP cleavage, a marker of cell death (91), without significant changes in cell cycle regulators or DDR factors, such as the phosphorylation of p53, Chk1 and Chk2, and p21 levels (**Figure S4A**). In all conditions, Caspases 3/8/9 presented already a low degree of cleavage, which was slightly increased in the case of Caspase 3 in XG19 and Caspases 3/9 in XG2 by melphalan and ML216 combination (**Figure S4B**). Thus, our data suggest that combination of BLM inhibition with melphalan induces cell death (**Figure 7**), likely through DNA damage accumulation above the cell’s tolerance level.

**Figure 8:**
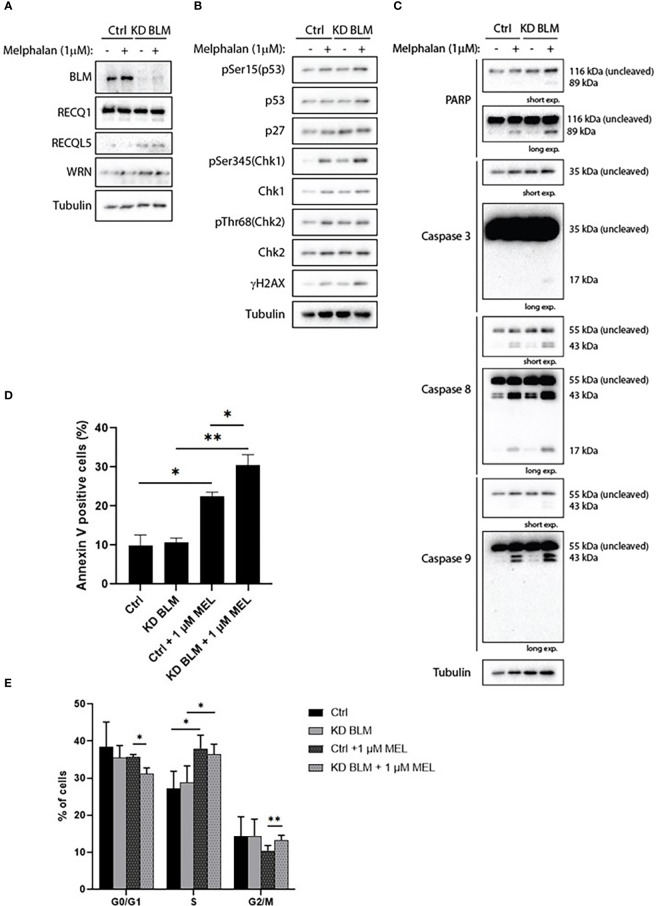
BLM depletion sensitizes MM cells to melphalan. XG2 cells were transduced with control lentiviruses (Ctrl) or miRNA against BLM to stably knock-down its expression (KD BLM). Both cell lines were treated with 1 μM melphalan for 96 hours and samples were collected to analyze **(A)** BLM depletion and protein levels of the other RECQ helicases; **(B)** cell cycle and DDR markers by western blot; **(C)** PARP and caspases cleavage as apoptosis markers by western blot; **(D)** apoptotic cells (Annexin V+) by flow cytometry; **(E)** cell cycle distribution using BrdU incorporation and DAPI staining as in **Figures 5** and **7**. Asterisks indicate a significant difference after applying a Student’s t-test for pairs. * p-value < 0.05, ** p-value < 0.01. All experiments in this figure were repeated 3 times independently.

In order to confirm the specificity of our results with ML216, we transduced the XG2 cell line with lentivirus containing specific miRNA to knock-down BLM expression (KD BLM). We validated the depletion of BLM protein by western blot, and confirmed that BLM downregulation did not significantly affect the level of expression of the other RECQ helicases (**Figure 8A**). XG2 control and KD BLM cell lines were treated with 1 μM melphalan for 4 days, and the DDR and caspases activation were analyzed by western blot. In agreement with our results with BLM inhibition in the XG2 parental cell line (**Figure 7** and **Supplementary Figure S5**), the treatment with melphalan induced DDR activation (**Figure 8B**) and more PARP and caspases cleavage (**Figure 8C**) in KD BLM cells than in control cells. Moreover, apoptosis level was higher in KD BLM in response to melphalan (**Figure 8D**). The treatment with the alkylating agent also induced an accumulation of cells in S-phase both in control and KD BLM cells, with small but significant differences also in the repartition of G0/G1 and G2/M populations (**Figure 8E**). These results validate the specificity of the phenotypes obtained with the chemical inhibition of BLM with ML216 in combination with melphalan, and strengthen the notion that BLM activity and levels are important for the response to DNA damage agents in myeloma cells.

**Figure 9:**
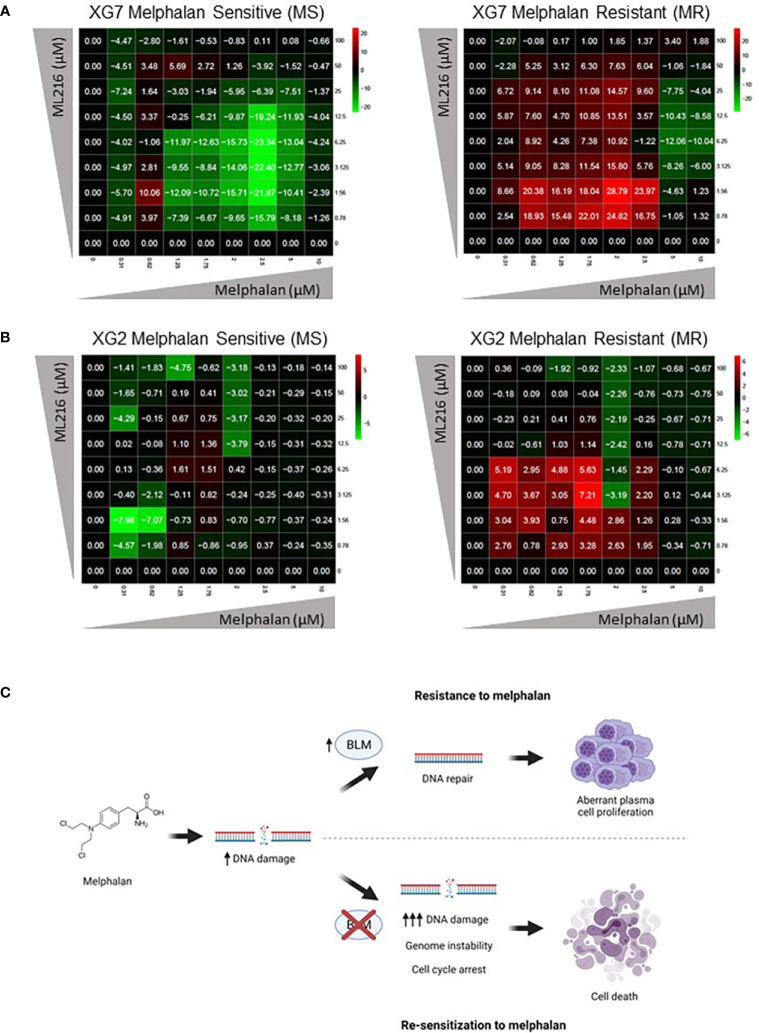
ML216 re-sensitizes Melphalan-resistant MM cells to Melphalan. Dose-response matrixes to measure synergy of ML216 and Melphalan co-treatment. Synergy scores are shown using a continuous pseudo-color scale ranging from dark-green (=antagonism) to dark-red (=synergism). **(A)** XG7 parental cells and XG7 MR (Melphalan Resistant) (Melphalan IC50: 0.625 μM and 7.5 μM respectively) were treated for 4 days with the indicated doses of ML216 and melphalan. Cell viability was assessed using CellTiter-Glo Luminiscent Cell Viability Assay and was normalized with respect to untreated conditions. Matrixes show the mean of 3-4 independent experiments. **(B)** Same as in **(A)** but comparing XG2 parental and XG2 MR (Melphalan Resistant) (Melphalan IC50: 0.625 μM and 2 μM respectively). Matrixes show the mean of 3 independent experiments. **(C)** Treatment of MM cells with melphalan produces DNA damage. Cells that overexpress BLM can cope better with the drug-induced DNA damage and therefore survive to the treatment, showing a resistant phenotype. On the contrary, BLM inhibition in combination with melphalan increases DNA damage to levels that tumoral plasma cells cannot efficiently repair, leading to cell cycle arrest and eventually to cell death, overcoming melphalan-resistance. Figure was created with BioRender.com.

Finally, based on our previous observation of a synergy between ML216 and melphalan (**Figure 6**), we hypothesized that the inhibition of BLM could increase the sensitivity of cells to melphalan, notably in melphalan-resistant MM cells, which would be of therapeutic interest. We explored this possibility by comparing the sensitivity of XG2 and XG7 cell lines to their melphalan-resistant counterparts (XG2 MR and XG7 MR) (70). Interestingly, ML216 synergized with melphalan to inhibit cell growth specifically in XG2 and XG7 melphalan-resistant cell lines (**Figures 9A, B**). These data indicate that BLM inhibition could represent a new therapeutic option to overcome resistance to melphalan in MM cells (**Figure 9C**). A mutational signature (named SBS-MM1) linked to exposure to high-dose melphalan in MM patients and related to relapse has been described (92–94). These mutations mostly occur in the late-replicating and non-coding parts of the genome (92). In addition, another work reported that *TP53* mutation or loss are linked to melphalan resistance and that inactivation of DDR genes such as *ATM*, *FANCA*, *RAD54B*, and *BRCC3*, enhances the response to the treatment (95), which argues in favor of the combination of BLM inhibition with melphalan to increase melphalan effect. We compared the mutational burden of both XG2 (*TP53* mutated) and XG7 (*TP53* wild type) melphalan-resistant cell lines to their parental counterparts and identified 16 mutated genes (**Supplementary Table S2**) common to both melphalan-resistant cell lines. Four of those genes, *TBP* (TATA-binding protein) (96), *TRRAP* (Transformation/transcription domain-associated protein) (97), *CTBP2* (C-terminal-binding protein 2) and *CTDSP2* (Carboxy-terminal domain RNA polymerase II polypeptide A small phosphatase 2), play a role in transcription regulation, either directly or indirectly, which suggests that resistance to melphalan may be mediated not only by mutation of genes involved in DDR, but also by altered regulation of this and other pathways due to changes in transcription. In addition, *TRRAP* and *CTBP2* also play roles in DNA repair. On the one hand, TRRAP, a member of the PIKK family as ATM, has been involved in the regulation of DDR by mediating DSB repair (98) and we have recently reported that its mutation is associated with high-risk MM (99). On the other hand, CTBP2 has roles in diminishing cell cycle arrest and BRCA-mediated DDR, and in apoptosis regulation (100). Thus, in future studies, it would be interesting to further analyze the implications of *TRRAP* and *CTBP2* mutations in the development of resistance to melphalan in MMCs.

## Discussion

During the past decades, the development of new therapies has significantly prolonged the survival of MM patients. However, resistance to chemotherapy and relapse remain frequent causes of death (101). The DNA alkylating agent melphalan is one of the main anti-myeloma treatments, alone or in combination with other drugs. Resistance to DNA damaging agents like melphalan could be caused by deregulation or mutation of DDR pathways or increased antioxidant defenses among others (58, 60, 61, 102–106). We have previously demonstrated that RECQ1, a DNA helicase important for the response to replication stress, has a role in cell survival to replication problems and is related to drug resistance in MM cells (61). Another helicase from the RECQ family, namely BLM, is a DDR factor necessary for correct HR, whose mutations are associated with the cancer-prone Bloom’s syndrome (27). In this study, we present evidence that the DNA helicase BLM is also associated with MM cell survival and resistance to DNA damaging chemotherapy. We found that *BLM* expression increases along the progression of the disease and that *BLM* is differentially expressed among MM patients, with high *BLM* expression associated with a bad prognosis (**Figures 1**, **2**).

It has been proposed that BLM can act both as a tumor suppressor and as a proto-oncogene. On the one hand, *BLM* loss or mutation leads to increased genetic instability and BS development, which points to a role as a tumor suppressor. On the other hand, increased *BLM* expression has been associated with multiple types of cancers, suggesting a proto-oncogenic function [reviewed in (107)]. For example, a recent study reported a correlation between *BLM* overexpression and poor overall survival in lung and gastric cancer patients (108). Similarly, our data showed that high *BLM* expression correlates with worse overall and event free survival in MM patients (**Figure 2**), confirming a proto-oncogenic role in MM as well. High levels of *BLM* were also found in hematological malignancies such as myeloid leukemia, lymphoma and myeloma (62, 109). Furthermore, transcriptomics analysis of fibroblasts from BS patients identified cell proliferation and survival genes, as well as immunological pathways, as the topmost deregulated in this disease (110, 111). Similarly, our GSEA results showed an increase in the expression of proliferation-associated genes in MM patients with high level of *BLM* expression and bad prognosis (**Figure S1**).

We took advantage of model MM cell lines derived from patients to further characterize the role of BLM in MM. A panel of HMCLs showed different responses to the BLM inhibitor ML216, with IC50 concentrations ranging from 1.3 μM for the most sensitive cells to 16.9 μM for the most resistant one. Intriguingly, sensitivity to ML216 did not correlate with the levels of expression of any tested RECQ helicase (BLM, RECQ1, RECQL5, and WRN), the cell lines’ basal DDR activation, their MM molecular subgroup or mutations in several oncogenes (**Figure 3**). Loss of a particular DDR pathway in cancer cells can make cells more dependent on other pathways. Thus, one possibility is that deregulation of other molecular factors, likely involved in DDR and/or cell cycle regulation, could account for the differential sensitivity to BLM inhibition. However, BLM has other cellular functions other than DNA repair (reviewed in [80)], that could also be responsible for the differences in the response to ML216. For example, in physiological conditions, BLM is required for fork progression and stability, resolution of R-loops or ultrafine anaphase bridges, and acts during replication stress by unwinding unusual DNA structures. BLM also regulates the correct replication of telomeric regions and BLM-deficient cells show a slowdown of replication forks and an increase in G4 at telomeres (52). In addition, BLM has been reported to facilitate pre-mRNA synthesis by direct binding with RNA Pol I and DNA topoisomerase I (82, 112, 113). Thus, inhibiting BLM would also affect ribosome biogenesis, which is known to be of particular importance for fast-proliferating cells such as tumor cells (114). Finally, it has been suggested that BLM would regulate transcription of a set of genes *via* its interaction to their G4 motifs (110). Therefore, given the plethora of molecular mechanisms in which BLM is involved in the cells, it is likely that the different sensitivity to ML216 inhibitor shown by our panel of MM cell lines would depend not only on each cell line tolerance to DNA damage, but also to alterations in the other BLM-regulated processes.

We demonstrated that continuous BLM inhibition induces cell cycle arrest and eventually leads to apoptosis in HMCLs. Importantly, this toxicity seems specific to myeloma cells, since ML216 poorly affected non-myeloma primary cells from patients (**Figure 4B, C**). This is likely due to the role of BLM as a safeguard of genomic stability, making rapidly dividing tumoral cells more dependent on its activity to cope with DNA damage and replication-transcription conflicts. This notion provides a strong rationale to combine BLM inhibition with DNA damaging agents, in order to overload the cell with DNA damage while impairing its repair. Indeed, our data show that ML216 potentiated the effect of melphalan to kill the 3 tested HMCLs (**Figure 6**). Double treatment induced PARP and caspases cleavage, concomitant with a strong cell cycle arrest and increased cell death, supporting the idea of causing a DNA damage overload to kill tumor cells (**Figure 9C** and **Supplementary Figure S5**). A recent work has reported a synergy between ML216 and the PARP inhibitor olaparib with irradiation to kill NSCLC (non-small cell lung cancer) cells by inhibiting HR and promoting NHEJ (115). Therefore, the interest of the combination of ML216 with chemotherapies currently in clinical use deserves further investigation.

Resistance to drugs remains a major concern in the therapeutic management of MM. We have previously reported that *BLM* expression correlates with sensitivity to the immunomodulatory agent lenalidomide, a standard-of-care drug in MM (23). Interestingly, we have found a synergy between ML216 and lenalidomide in HMCLs (**Supplementary Figure S3B**). However, the mechanism of this synergy remains to be explored, since no significant effect on IMiD targets including Myc and IRF4 levels was observed upon treatment with ML216 (**Supplementary Figure S4C**). Melphalan is another standard-of-care drug used both in patients eligible and non-eligible for autologous hematopoietic stem cell transplantation, alone or in combination with other chemotherapeutic agents, respectively (56, 116, 117). However, patients often acquire mutations in DDR pathways that result in resistance (58, 60). By using melphalan-resistant cell models, we showed that inhibition of BLM may be a good strategy to overcome such resistance (**Figure 9A, B**). Similarly, we previously reported that sublethal concentrations of the inhibitors of Chk1 (AZD7762) and Cdc7-Dbf4 (XL413), both kinases involved in DNA damage and replication stress response, overcome resistance to melphalan in HMCLs (70). Thus, the combination of DDR inhibitors and DNA damaging agents should be further explored as a therapeutic option for MM patients with resistance to DNA damage chemotherapies.

Targeting DDR factors to enhance sensitivity to melphalan in MM has already been proposed. In particular, treatment with bortezomib reduced the levels of Fanconi Anemia (FA) factors BRCA1/2 and FANCD2, and proved effective to induce DNA damage and cell death in combination with melphalan (103). FA is an autosomal recessive disorder characterized by genetic instability that leads to developmental abnormalities, BM failure and elevated risk of developing certain types of cancer like acute myeloid leukemia and squamous cell carcinomas (118, 119). BS and FA disease present some overlapping phenotypes, like predisposition to cancer and immunodeficiency, and are both characterized by genetic instability. Although their genetic origins are different, it has been proposed that the BS complex (BLM, RMI1, RMI2 and Topoisomerase III-a) and the FA pathway are related by protein interactions, forming the BRAFT multiprotein complex (BLM, RPA, FA and Topoisomerase III-α), which has various DNA-processing activities (120, 121). In addition, BLM interacts with the helicase FANCJ (122), the DNA translocase FANCM interacts with FANCF, RMI1 and Topoisomerase III-a, linking both complexes (123). Moreover, BLM has been shown to promote the activation of the FA pathway through FANCD2 in response to cross-linking agents (124). Thus, it is not surprising that BLM inhibition also increases the toxicity of melphalan.

The notion to combine DNA damaging agents with drugs that target DDR pathways is gaining attention as a potential way to increase anticancer therapy effectiveness. For instance, inhibitors of RAD51 and WRN sensitize cancer cells to DNA damaging agents (125, 126). Thus, development of new inhibitors that target other DDR factors to be used in combination with current chemotherapeutic drugs becomes an appealing option to treat cancer more efficiently and confront drug resistance. BLM, a critical proven factor for genomic stability maintenance, is therefore an interesting candidate for combination therapies. Indeed, several experimental and computational studies have proposed RECQ helicases as potential targets in a range of different cancers (127–130). However, till recently only the small molecule ML216 had been developed to target BLM, which is still poorly characterized in treating cancer cells. Of note, isaindigotone and quinazolinone derivatives have been recently reported as potential new BLM inhibitors. Both inhibit proliferation and trigger apoptosis and DDR activation in the human colon cancer cell line HCT116 (131, 132). Also recently, a screen for antiproliferative drugs in breast cancer identified HJNO, a tetrandrine derivative which inhibits BLM DNA binding, unwinding and ATPase activities, diminishing breast cancer cells proliferation (133). Another study has analyzed derivatives of ML216 that seem highly specific allosteric inhibitors of BLM, that could be used to cause highly cytotoxic BLM-DNA complexes to kill cancer cells (134). In the next years, further work to develop and characterize new BLM inhibitors is warranted.

In MM, most patients develop resistance to the existing therapies, including melphalan. Our data suggest that *BLM* expression can be a good biomarker for MM and that combination of BLM inhibitors with DNA damaging drugs could be of therapeutic interest to treat MM patients who have developed resistance to melphalan. It is important to keep in mind that chemotherapy drugs used to target tumor cells are also toxic to other types of healthy cells, leading to toxicity and, ultimately, the development of secondary cancers in many patients later in life. Several mechanisms associated with MMC resistance to genotoxic treatments have been described, underlining the myeloma endemic heterogeneous landscape (135). These findings provide several therapeutic strategies to overcome drug resistance and limit mutagenic effects of genotoxic agents in MM (135). Exploiting synthetic lethality between DNA repair inhibitors and DNA-damaging agents would allow lower concentrations of the latter to be used, limiting undesirable side effects. The clinical manifestations of patients with Bloom’s syndrome indicate that BLM activity is crucial for the maintenance of genetic stability at the organismal level. However, the toxicity associated with BLM inhibition in the context of therapeutic treatment, i.e. inhibition that is not sustained over time for years, is not likely to have such a dramatic impact on the fitness of cancer patients compared to patients with BS. Yet, BLM inhibitor’s toxicity needs to be carefully addressed using *in vivo* models to assess the benefits and risks of its use in cancer treatment.

## Data availability statement

The datasets presented in this study can be found in online repositories. The names of the repository/repositories and accession number(s) can be found below: https://www.ebi.ac.uk/arrayexpress/, E-MTAB-37, https://www.ebi.ac.uk/arrayexpress/, E-TABM-937, https://www.ebi.ac.uk/arrayexpress/, E-TABM-1088.

## Author contributions

SO performed the research and wrote the paper. EV, LD and JD participated in the research and in the writing of the paper. MJ, GR, NR and HB participated in the research. EA participated in biocomputational analyses. AS, DH, LV and CH participated in clinical data analysis and participated in the writing of the paper. Y-LL, JB, AC and PP participated in the research and in the writing of the paper. JM supervised the research and the writing of the paper. All authors contributed to the article and approved the submitted version.
