# Supplementary material for: The BLM helicase is a new therapeutic target in multiple myeloma involved in replication stress survival and drug resistance
Source: Front Immunol. 2022 Dec 9;13:983181. doi: 10.3389/fimmu.2022.983181 (PMC9780552; doi:10.3389/fimmu.2022.983181)
Supplement: Supplementary file 1 [file DataSheet_1.docx]

Supplementary Material

**Supplementary Figure S1. (A)** HMCLs used to analyze *BLM* expression in Figure 1A and their chromosome translocation (*Moreaux et al., 2011 Haematologica*). **(B)** Top gene set significantly associated with high *BLM* expression in MM**.** Gene Set Enrichment Analysis (GSEA) enrichment plots with the absolute enrichment p-value and the normalized enrichment score of the gene set. **(C)** Plasma cell labeling index (PCLI, % of S phase of MM cells) was investigated using BrdU incorporation and flow cytometry in a cohort of 89 patients at diagnosis. The correlation between *BLM* expression and PCLI was determined with a Spearman’s test.

**
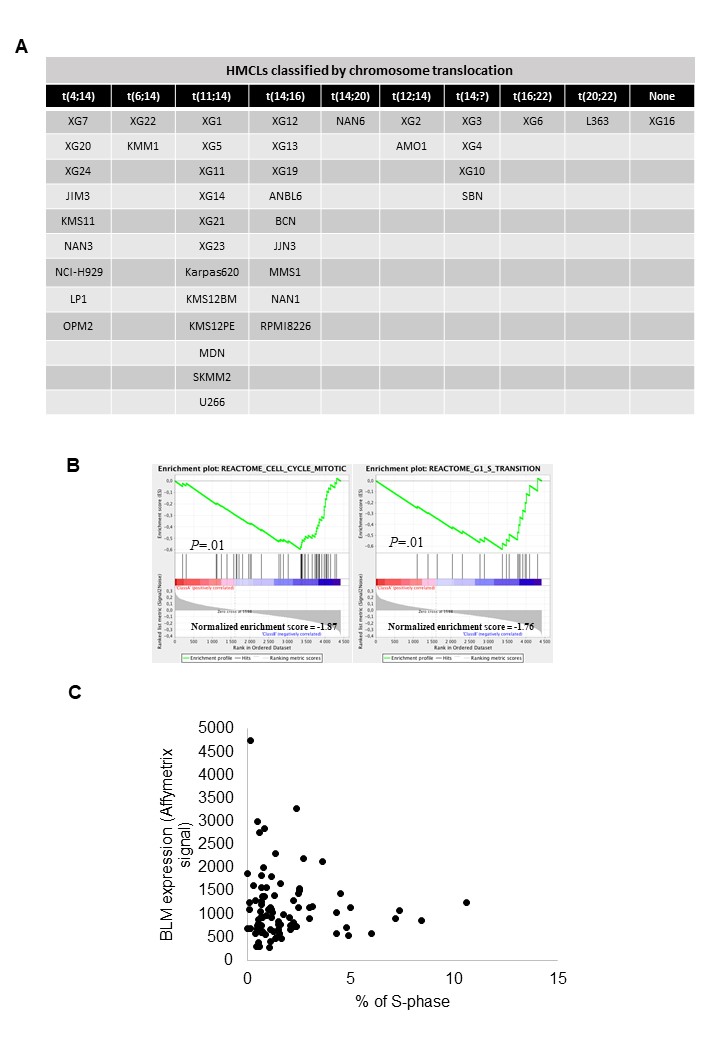
**

**Supplementary Figure S2. (A)** Comparison of BLM expression with Affymetrix GEP-based risk scores including RS, HRS, IFM and GPI. Statistical analysis was performed using a Wilcoxon test. NS = not significant.


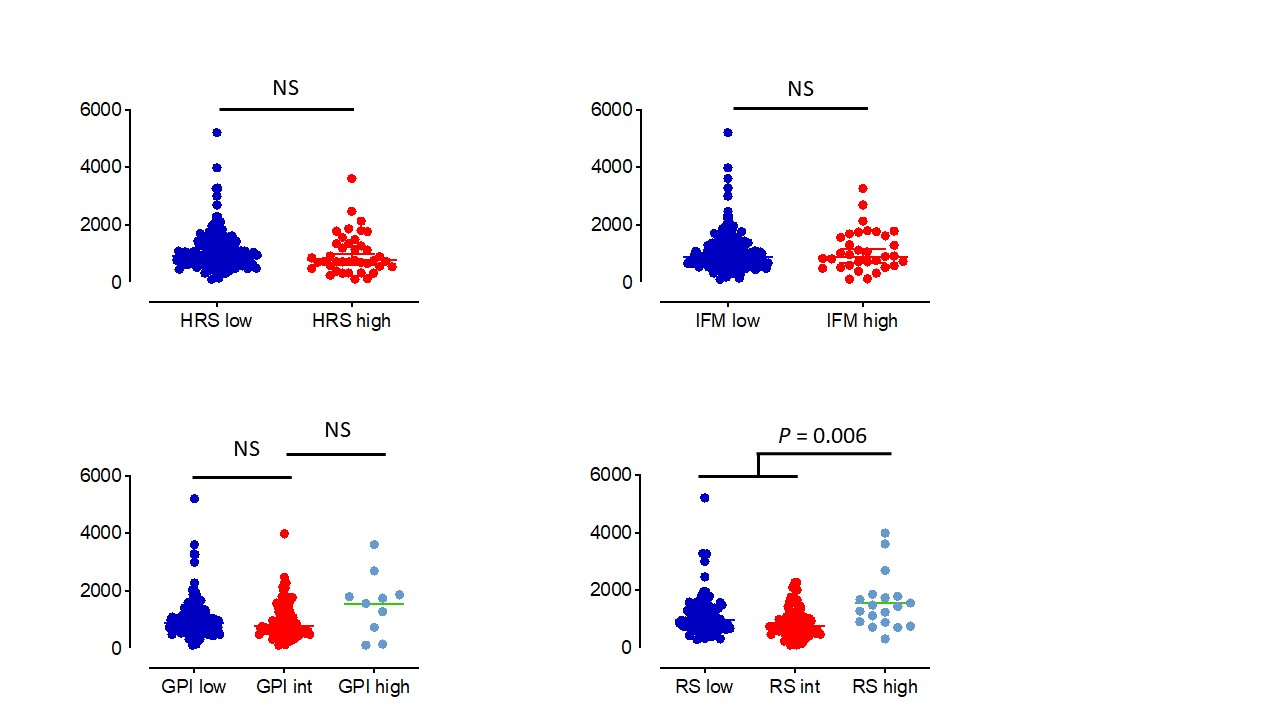
**A**

**Supplementary Figure S3. ML216 does not affect BLM protein levels or subcellular localization. (A)** XG19 and XG2 cells were treated with ML216 at the indicated doses during 48 or 96 hours. Samples were collected and BLM protein levels were analyzed by Western blot. **(B)** Immunofluorescence to detect BLM chromatin localization. XG19 and XG2 cells were treated with ML216 (1-3 μM, and 5-15 μM, respectively) (or the vehicle DMSO (mock) for 48 hours. Cells were deposited onto poly-lysine coated glass slides using a Cytospin. Soluble cell fraction was pre-extracted by incubation with CSK buffer prior to fixation with 4% PFA in PBS. BLM and nucleolin were detected with specific antibodies and DNA was stained with DAPI. Scale bar = 10 μm.

**
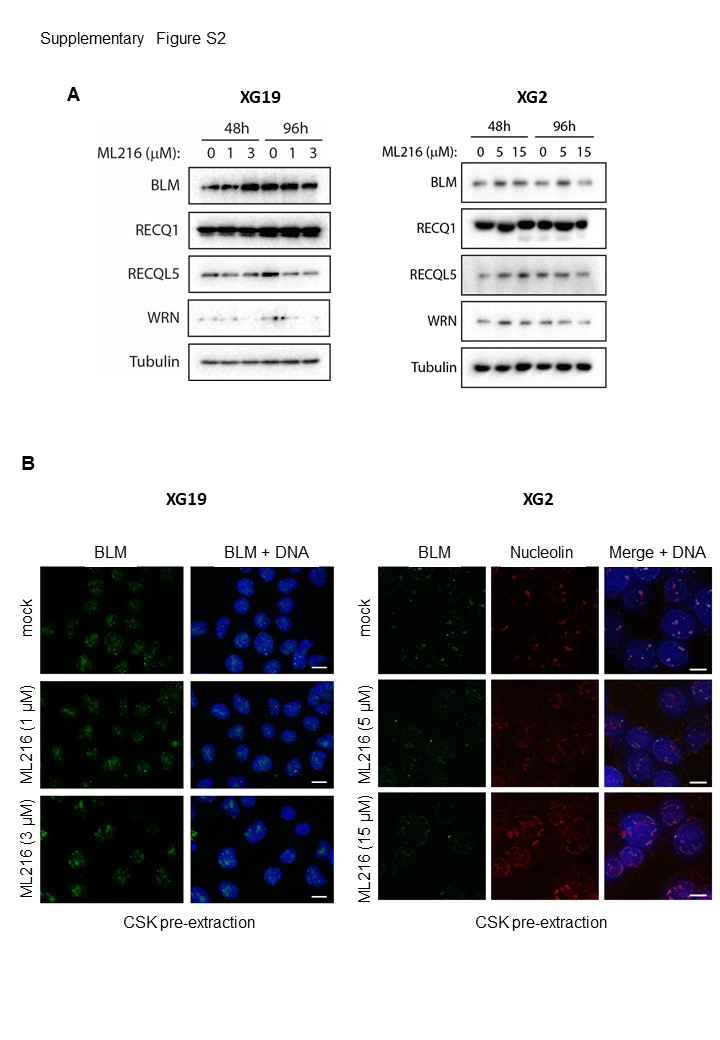
**

**Supplementary Figure S4. Synergy matrixes of ML216 combination with bortezomib and lenalidomide. (A,B)** Dose-response matrixes to measure synergy of ML216 and bortezomib or lenalidomide co-treatment. Synergy scores are shown using a continuous pseudo-color scale ranging from bright-green (=antagonism) to bright-red (=synergism). XG19, XG2 and XG1 were treated with increasing concentrations of ML216 (0.78125 – 25 μM), and of the proteasome inhibitor bortezomib (0.07813 – 10 nM), or the immunomodulatory drug lenalidomide (0.78125 – 50 μM) for 4 days. Cell viability was assessed using the CellTiter-Glo Luminiscent Cell Viability Assay and was normalized to untreated conditions. Matrixes show the average of 3 independent experiments. **(C)** XG2 cells were treated with ML216 (5 or 15 μM) for 48h and 96h and the protein levels of Myc and IRF4 were analyzed by western blot. Tubulin was used as a loading control. Figure shows one representative out of 3 independent experiments.

**
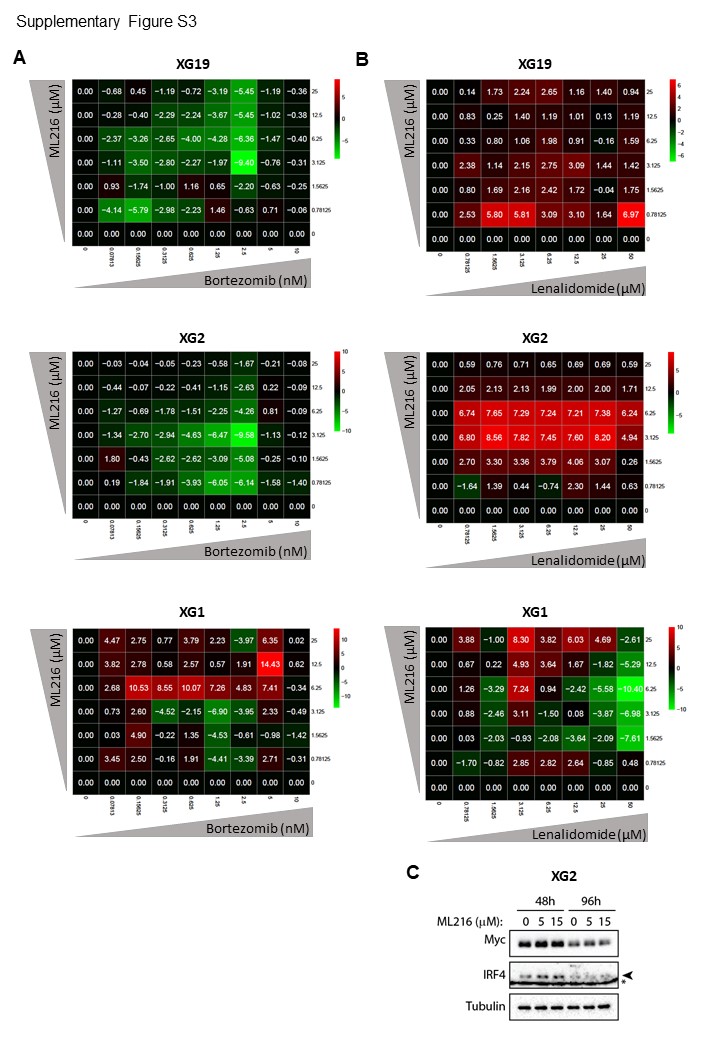
**

**Supplementary Figure S5. Analysis of cell cycle, apoptotic and DNA repair pathways in response to combination of ML216 and melphalan. (A, B)** XG19 and XG2 cells were treated with the indicated doses of ML216 and melphalan for 48 hours. Cells were collected and protein extracts were prepared to analyze cell cycle regulators, DDR factors and apoptotic pathways.


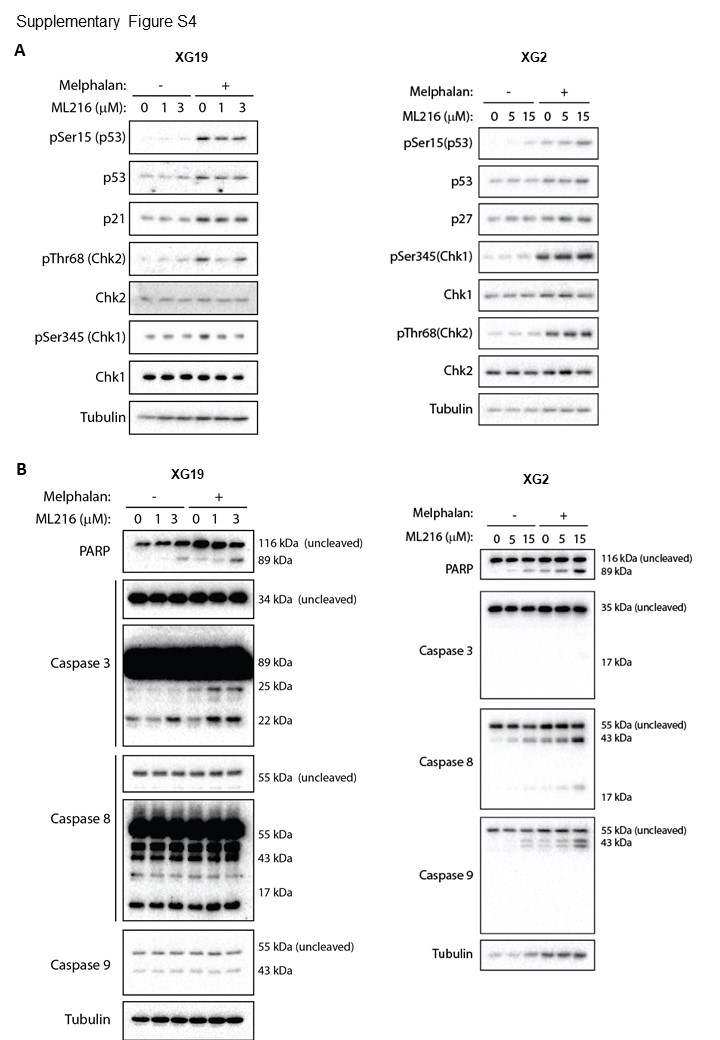


**Supplementary Table S1. Cox univariate and multivariate analysis of overall survival (OS) in HM cohort.**

| Univariate COX analysisAA2:D42 |  | HM-cohort | |
| --- | --- | --- | --- |
| Overall Survival |  | OS | |
|  | Pronostic variable | Proportional hazard ratio | *P*-value |
|  | BLM | 2.94 | 0.001 |
|  | ISS | 1.84 | 0.002 |
|  | B2m | 1.1 | <0.0001 |
|  | t(4 ;14) | 3.32 | <0.0001 |
|  | del17p | 3.44 | 0.02 |
|  | HRS | 2.37 | 0.01 |
|  | IFM score | 2.49 | 0.01 |
|  | GPI | 2.54 | <0.0001 |
|  | RS | 4.16 | <0.0001 |
|  |  |  |  |
| 2 by 2 Multivariate COX analysis |  | HM-cohort | |
| Overall Survival |  | OS | |
|  | Pronostic variable | Proportional hazard ratio | *P*-value |
|  | BLM | 2.77 | 0.001 |
|  | ISS | 1.75 | 0.005 |
|  | BLM | 2.98 | 0.001 |
|  | B2m | 1.1 | <0.0001 |
|  | BLM | 3.1 | <0.0001 |
|  | t(4 ;14) | 3.53 | <0.0001 |
|  | BLM | 3.28 | <0.0001 |
|  | del17p | 3.75 | 0.01 |
|  | BLM | 2.84 | 0.001 |
|  | HRS | 2.24 | 0.02 |
|  | BLM | 2.78 | 0.001 |
|  | IFM score | 2.23 | 0.04 |
|  | BLM | 2.45 | 0.006 |
|  | GPI | 2.27 | 0.001 |
|  | BLM | 2.05 | 0.03 |
|  | RS | 3.57 | <0.0001 |
|  |  |  |  |
|  |  |  |  |
| Multivariate COX analysis |  | HM-cohort | |
| Overall Survival |  | OS | |
|  | Pronostic variable | Proportional hazard ratio | *P*-value |
|  | BLM | 2.52 | 0.01 |
|  | ISS | 1.29 | NS |
|  | B2m | 1.1 | 0.02 |
|  | t(4 ;14) | 3.12 | 0.01 |
|  | del17p | 2.1 | NS |
|  | HRS | 1.26 | NS |
|  | IFM score | 0.42 | NS |
|  | GPI | 0.78 | NS |
|  | RS | 3.51 | 0.001 |

# Supplementary Table S2. Genes mutated in both melphalan-resistant XG2 and G7 and not in their parental counterparts. XG2 and XG7 cell lines chronically exposed to melphalan to develop resistance were sequenced to find new mutations caused by the treatment (see *de Boussac et al., 2020. Haematologica* for details). The 16 genes commonly mutated in both melphalan-resistant cell lines after chronic exposure to melphalan are listed.

| GENE | PROTEIN |
| --- | --- |
| *HTT* | Huntingtin |
| *ZSWIM6* | Zinc finger SWIM domain-containing protein 6 |
| *HGC6.3* | HGC6.3 protein |
| *TBP* | TATA-box-binding protein |
| *TRRAP* | Transformation/transcription domain-associated protein |
| *MUC3A* | Mucin-3A |
| *AGAP3* | Arf-GAP with GTPase, ANK repeat and PH domain-containing protein 3 |
| *FDFT1* | Squalene synthase |
| *SVEP1* | Sushi, von Willebrand factor type A, EGF and pentraxin domain-containing protein 1 |
| *CYP26C1* | Cytochrome P450 26C1 |
| *CTBP2* | C-terminal-binding protein 2 |
| *MUC6* | Mucin-6 |
| *DCHS1* | Protocadherin-16 |
| *TAS2R46* | Taste receptor type 2 member 46 |
| *CTDSP2* | Carboxy-terminal domain RNA polymerase II polypeptide A small phosphatase 2 |
| *MMP17* | Matrix metalloproteinase-17 |
